# Pelvic Organ Prolapse: A Review of In Vitro Testing of Pelvic Support Mechanisms

**DOI:** 10.31486/toj.19.0089

**Published:** 2020

**Authors:** Cassandra K. Conway, Shelby E. White, Rachel Russell, Claire Sentilles, Gabrielle L. Clark-Patterson, Kristin S. Miller, Laurephile Desrosiers, Leise R. Knoepp

**Affiliations:** ^1^ Department of Biomedical Engineering, Tulane University, New Orleans, LA; ^2^ Department of Obstetrics and Gynecology, Division of Female Pelvic Medicine and Reconstructive Surgery, Ochsner Clinic Foundation, New Orleans, LA; ^3^ The University of Queensland Faculty of Medicine, Ochsner Clinical School, New Orleans, LA

**Keywords:** *Connective tissue*, *extracellular matrix*, *genitalia*, *pelvic floor*, *pelvic floor disorders*, *pelvic organ prolapse*

## Abstract

**Background:** Pelvic organ prolapse (POP) affects a significant portion of the female population, impacting quality of life and often requiring intervention. The exact cause of prolapse is unknown.

**Methods:** We review some of the current research that focuses on defining the elements involved in POP, with a focus on in vitro testing.

**Results:** Treatment for POP, ranging from physical therapy or pessary use to more invasive surgery, has varying success rates. This variation is, in part, because the pathophysiology of pelvic floor support—and thus dysfunction—is incompletely understood, particularly regarding the structural components and biomechanical properties of tissue. However, researchers are working to identify and quantify the structural and functional dysfunction that may lead to the development of this condition.

**Conclusion:** Given the limited understanding of prolapse development, more research is needed to quantify the microstructure of the pelvic organs and pelvic support structures, with and without prolapse. Identifying biomechanical properties in multiaxial configurations will improve our understanding of pelvic tissue support, as well as our ability to establish predictive models and improve clinical treatment strategies.

## PELVIC ORGAN PROLAPSE

Pelvic organ prolapse (POP) is a condition characterized by the descent of the pelvic organs, resulting in external protrusion of these organs through the vaginal introitus. Multiple scaffolding mechanisms maintain and prevent POP, including bony structures, muscle, and fascial and connective tissue. Prolapse is hypothesized to be a result of reduction in the mechanical integrity of pelvic organ support structures, such as the endopelvic fascia and levator ani muscles.^1^ Pelvic floor disorders can manifest in various ways, including urinary and defecatory dysfunction, urinary and fecal incontinence, sexual dysfunction, and physical discomfort. The constellation of issues that arise from pelvic floor disorders can cause psychological distress that leads to poor quality of life.^2-4^ Because most women present with POP between the ages of 50 and 70 years, many women spend a large portion of their lives dealing with its effects.^5^ Approximately 40% of women between the ages of 50 and 79 years in the United States are estimated to be affected by some degree of POP, and as the elderly and obese populations continue to increase, the incidence of POP is also expected to increase.^6^ In 2001, POP accounted for an annual estimated cost of more than $1 billion in the United States.^7^ The impact of the cost of POP in the United States is expected to dramatically rise as the elderly and obese populations increase.^6^

### Pelvic Organ Prolapse Diagnosis and Treatment

The evaluation of POP begins with a detailed medical history and physical examination. The number of pregnancies, the type of delivery, the length of the second stage of labor, and history of any assisted delivery technique are some of the salient points to ascertain during history taking. The physical examination is typically performed in both the reclining and standing positions, at rest and with maximum Valsalva. A thorough examination of the external genitalia is recommended, followed by placement of a disarticulating bivalve speculum in the vagina, allowing for the evaluation of prolapse in all compartments (anterior, posterior, and apical) when separated. The POP quantification measuring system (POP-Q) is the most common system used to document physical findings of prolapse and is the only validated system. This system also allows for systematic and consistent staging of prolapse.^8-11^

Treatment of prolapse remains a challenge. Mild or less symptomatic cases of prolapse can be treated through conservative measures such as pelvic floor therapy and pessary use. Pelvic floor physical therapy can result in modest but measurable improvement in POP^12^ and can decrease the symptomatology (eg, pelvic pressure and heaviness) often experienced with prolapse.^13^ Patients with POP can also be fitted with pessaries, reusable vaginal inserts used to elevate POP above the vaginal opening.^14^ Although many patients can initially be successfully fitted with a pessary,^14^ certain factors, including severe posterior vaginal prolapse, development of occult stress urinary incontinence with pessary use, and desire for surgery at a patient's first visit, are associated with increased likelihood of discontinuing pessary use.^15-16^

Pelvic reconstructive surgery is indicated for women with symptomatic POP who have failed conservative management or desire surgical intervention,^17^ and 10% of women with normal life expectancies will undergo surgery for prolapse.^3^ Surgical options to treat prolapse can be categorized as (1) reconstructive, (2) compensatory or augmented, or (3) obliterative.^5^ Reconstructive procedures use native tissue to restore anatomy (eg, anterior/posterior colporrhaphy, uterosacral ligament suspension); compensatory procedures use biologic graft or synthetic mesh to augment native tissue (eg, sacrocolpopexy); and obliterative procedures partially or totally close the vagina (eg, colpocleisis). Each approach has advantages and disadvantages, and all surgical corrections aim to provide long-term success. However, sustainability has been a challenge thus far, with reoperation rates as high as 30% by the age of 79 years.^3^

## PELVIC FLOOR BASIC ANATOMY

**Figure 1.**
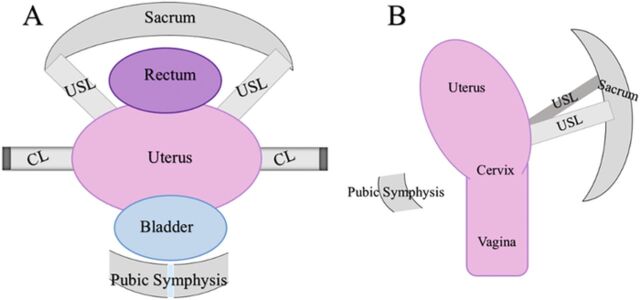
**(A) Coronal (frontal) depiction of normal pelvic floor anatomy: pubic symphysis, bladder, uterus, rectum, sacrum, uterosacral ligaments (USL), and cardinal ligaments (CL). (B) Sagittal depiction of the same anatomy—now including the vagina—that clarifies the location of the USLs. Failure of the pelvic support ligaments, such as the CLs and USLs, has been implicated as a potential factor in development of pelvic organ prolapse.^18,37-39^**

The anatomy of the female pelvic floor consists of pelvic organs, musculature, bony structures, and various connective tissues. The pelvic organs include the bladder, uterus, vagina, bowel, and rectum, which are seated within the constructs of the bony pelvis (Figure 1).^18^ The bony pelvis consists of 2 innominate bones, both fused to the sacrum posteriorly and to each other anteriorly at the pubic symphysis. Each innominate bone is composed of the ilium, ischium, and pubis.^19^

The urinary bladder is the most anterior of the pelvic floor organs, with the uterus resting posteriorly to it. The inferior-most part of the uterus is the cervix, and the vagina is caudal to these structures. The rectum sits posteriorly to the uterus, cervix, and vagina. Each of these structures consists of smooth muscle and connective tissue layers.^11,20-30^ The major muscles comprising the pelvic floor include the levator ani muscles (pubococcygeus, puborectalis, and iliococcygeus), the coccygeus muscle, the obturator internus muscles, the deep and superficial perineal muscles, the external anal sphincter, and the striated urethral sphincter, all of which are lined with endopelvic fascia.^31,32^ The pubococcygeus muscle is the bulkiest and medial-most portion of the levator ani complex that arises from the posterior pubis and anterior portion of the arcus tendineus fascia pelvis (ATFP). The ATFP, the origin of the levator ani muscles, is a dense connective tissue structure that spans the area between the pubic ramus to the ischial spine. Bilaterally, the obturator internus muscles are lateral and anterior to the ATFP.

The major functions of the pelvic floor muscles are supporting the pelvic organs and viscera, sustaining intrapelvic pressure forces, and controlling urinary and fecal continence through sphincter mechanisms.^31^ The endopelvic fascia, a network of connective tissue, envelops the pelvic organs and loosely connects them to the more supportive bony and musculature structures. The fascia helps to support the vagina and uterus and allows for mobility and storage of urine and stool.^33^ Weakening of these pelvic floor muscles or disruption of the endopelvic fascia can lead to destabilization of the pelvic organs, possibly resulting in prolapse.

## PATHOPHYSIOLOGY OF SUPPORT FAILURE

Levator ani injury is a potential mechanism of POP.^20,34^ However, evidence of direct causality is limited.^35^ Tissue degradation and a corresponding weakness of certain endopelvic structures, such as the uterosacral ligament and the cardinal ligament, are also associated with POP. Accordingly, 3 levels of connective-tissue support of the vagina have been described by DeLancey,^36^ with the cardinal and uterosacral ligaments providing the strongest, level 1 (apical) support.^37-39^

Although the changes in gross anatomy related to development of POP have been reasonably characterized, investigations into the role of microstructural constituents in POP development are needed. Native tissue repair failure rates approach 40% within 2 years of the initial procedure.^40^ Concrete assessment of surgical outcome can be challenging, but the surgical failure rate, particularly with regard to the anterior compartment, remains high. Repair of POP using native tissue relies on the physiologic integrity of the reproductive tissue. However, if the tissue is compromised by prolapse and unable to mimic normal structure and function without considerable failure rates, other methods for repair are necessary.^41^ For example, to fortify and support native tissue, biologic or synthetic meshes are used in one-third of all prolapse reconstructive surgeries.^40^ Despite augmentation with synthetic mesh in 33% of prolapse surgeries, more than 30% of these procedures will have complications, including mesh extrusion, mesh erosion, pain, or compensatory failure of the posterior compartment, that often require a second procedure.^3,40-42^ Complications with synthetic meshes may be attributed to the stiffness of the implanted meshes, as increased rigidity can result in shear stresses or stress shielding on the surrounding tissues. Stress shielding is defined as a material buffering a tissue against normal loads. Thus, without exposure to normal mechanical stimulation, the supplemented native tissue begins to degenerate and may further contribute to a decline in overall tissue strength.^43^

The biomechanics of pelvic tissues is an understudied field. The extracellular matrix is the principal structural aspect of vaginal and pelvic ligament tissue, providing strength and elasticity.^44^ Therefore, a change in extracellular matrix composition may lead to an alteration in mechanical properties, which may lead to POP. Alterations in extracellular matrix structure resulting in decreased tissue function and integrity may help explain why subjects with connective tissue disorders have increased rates of prolapse.^45^ In vitro experimental methods can be used to determine the role of microstructural constituents in the function of healthy and diseased tissue.^46,47^ Combining traditional testing methods that rely on histology or immunohistochemistry with mechanical testing can create opportunities to evaluate the microstructural transformations that arise from pelvic floor dysfunction and help create predictive mathematical models to guide patient-specific treatments for prolapse in the future.^47^

## VAGINAL BIOMECHANICS

The vagina is essentially a fibromuscular tube with rugated folds that extends from the hymenal ring to the cervix. The vaginal canal is the external opening for the female reproductive system and plays an important role in intercourse, menstruation, and childbirth.^48^ The vaginal canal also protects the internal reproductive organs from infection and injury and is a support structure for the pelvic organs.^48^ The vaginal wall is composed of 4 layers: epithelium, subepithelium, muscularis, and adventitia. The epithelial layer consists of nonkeratinized, stratified squamous epithelium that responds to changes in the hormonal cycle and, in conjunction with cervical secretions, maintains the microbiome of the vagina.^48^ The subepithelium is a layer of dense connective tissue composed principally of collagen and elastin. The muscularis is comprised of smooth muscle fibers that are oriented in longitudinal and circumferential directions to provide support and to control the contractile response of the vagina.^49^ The adventitia is loose connective tissue that provides limited vaginal support but creates a conduit around the urinary bladder and rectum through which nerves, blood vessels, and lymph channels course.^48^ Overall, the vagina is a fibromuscular organ comprised principally of collagen fibers, elastic fibers, and smooth muscle.^50,51^

The extracellular matrix constituents of the vagina maintain structural integrity and facilitate daily function. The extracellular matrix is present throughout the vaginal wall, but the subepithelium and the muscularis contribute most of the passive support for the organ. Collagen and elastin are significant constituents of vaginal wall mechanical function. Collagen imparts high tensile strength, allowing the tissue to dynamically withstand significant load, and is the most abundant fibrous protein within the vaginal extracellular matrix.^47^ Elastin provides resilience and recoil to the tissue, allows the tissue to retain its shape, and also permits long-range deformability.^47^ Elastic fibers are important for a tissue's residual stress (the stress that exists within a tissue when all external loads are removed) and residual strain (the contribution of residual stress in the unloaded state of a tissue).^52,53^ Women with POP have tissue with lower elastic fiber content,^54^ and genetically modified mice with elastogenic deficiencies develop prolapse spontaneously (fibulin-5 knockout) or after experiencing vaginal birth (LOXL1 knockout).^55-57^ Thus, elastic fibers may be an important therapeutic target for POP treatment. In vitro experimental methods have been developed to determine the role of tissue extracellular matrix constituents.^46,47,58^ Through use of these techniques, further understanding of the etiology of prolapse may be determined and applied to clinical practice.

### Vaginal Tissue Experimental Methods and Mechanical Properties

**Figure 2.**
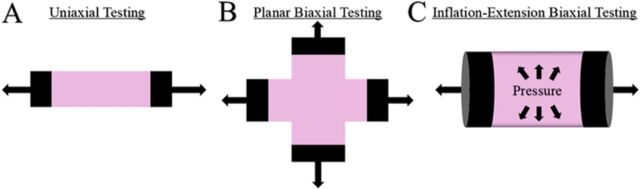
**Depictions of common in vitro mechanical testing configurations. (A) Uniaxial testing requires strips of tissue to be removed from the body. The tissue is clamped on both ends and pulled at constant rates through an electrical motor to imitate mechanical loads in one axis.^59-62,67,68^ (B) Planar biaxial testing techniques typically use tissue cut into a cruciform shape to incorporate loading of the tissue along a second axis.^70^ Biaxial testing is valuable in understanding the multiaxial response of tissues, as tissue structure usually has a preferred fiber direction. (C) Inflation-extension biaxial testing mimics the geometry of cylindrical organs such as blood vessels and invokes a multiaxial response by stretching the tissue axially, as well as pressurizing the tissue at set rates.^50,71^**

Uniaxial testing is an experimental method commonly used for soft tissues, such as tendons, to investigate biomechanical properties and improve understanding of tissue at a fundamental level (Figure 2).^59-62^ Rubod et al adapted a uniaxial technique for vaginal tissue from a ewe animal model.^63^ Ewes are beneficial models for prolapse, as they develop prolapse with age or after vaginal birth. The researchers tested samples of vaginal tissue from ewes under multiple experimental conditions to determine the best parameters for reproducibility in the testing protocol, investigating deformation rate, temperature, freeze-thaw cycles, and location in the vagina. Interestingly, freezing vaginal tissue did not significantly change the passive mechanical properties of the tissue. Additionally, in the ewe model, the anterior vaginal wall did not show significantly different mechanical properties than the posterior vaginal wall.^63^ Subsequently, Rubod et al compared the biomechanical properties of nonprolapsed human pelvic organs, including the vagina, bladder, and rectum.^64^ Adapting the uniaxial tensile tests established in their previous study, the researchers used cadaveric pelvic tissue to demonstrate the Mullins effect (change in mechanical properties of rubber-like materials resulting from the first extension) in the tissues through cyclic loading. They also compared posterior vs anterior wall integrity in the transverse and longitudinal directions. The vagina was stiffer than either the bladder or rectum in both the longitudinal and transverse directions, a finding that was attributed to the functions of the bladder and rectum. Because the bladder and rectum play a role in storing waste and releasing waste in a cyclic fashion, excessive rigidity of these tissues could prevent normal, dynamic function of these organs, such as complete emptying of urine or stool.^64^ Prompted by the experimental methods of Rubod et al, Jean-Charles et al adapted a protocol to investigate the mechanical properties of prolapsed and nonprolapsed vaginal tissue.^41^ Prolapsed tissue was less pliable in both the anterior and posterior vagina compared to nonprolapsed tissue. The researchers hypothesized that the increased stiffness in prolapsed tissue could be a factor in the failure of native tissue prolapse repairs.^41^ In the Chinese population, Lei et al investigated the biomechanical properties of vaginal tissue of premenopausal and postmenopausal women, based on a protocol to test skin samples on uniaxial tension.^65,66^ The study included 4 groups: premenopausal and postmenopausal control groups and premenopausal and postmenopausal groups with prolapse. Tissue was less elastic and stiffer in the premenopausal and postmenopausal prolapse groups compared to the premenopausal and postmenopausal control groups. However, the study did not determine if factors such as age, body mass index, and parity were directly causative of increased tissue stiffness.^65^ Peña et al also investigated the viscoelastic properties of prolapsed human vaginal tissue, incorporating uniaxial testing and mathematical modeling of the tissue to describe the response.^67^ Adding to data vigor, the researchers displayed modeling results of biological variability among subjects because tissue donors were geographically diverse and had various presenting pathologies. Study findings were limited, however, because Peña et al only tested the longitudinal response of the organ, even though the vagina experienced a multidirectional load in situ.^67^ In a continued investigation, Peña et al characterized the softening response of human vaginal tissue in both transverse and longitudinal directions.^68^ In findings similar to the human studies by Rubod et al, the vaginal tissue exhibited the Mullins effect: the tissue demonstrated an anisotropic response for both elastic and inelastic behavior.^64,68^

Moalli et al developed a method to test the entire vagina and supportive tissue complex in a rat model.^69^ Rat pelvises were removed en bloc from the body, and the legs and vertebrae were disarticulated from connecting joints. Clamped at either end, the tissue sample was stretched to the point of failure.^69^ The researchers concluded that the paravaginal support structures failed before the vagina itself, and therefore, the connective tissue support structures need to be studied further to better characterize prolapse.^69^ Using the same technique on oophorectomized young and middle-aged rats, Moalli et al described a loss in biomechanical properties of the pelvic support structures with the loss of native hormones in young animals and a restoration of properties for animals treated with hormones, suggesting that natural hormone loss may have a role in the deterioration of pelvic structures and subsequent development of prolapse.^1,69^

Huntington et al investigated the passive and active mechanical properties of the rat vagina in a planar biaxial configuration to study the anisotropic response (Figure 2).^70^ Circumferential properties were greater than longitudinal properties for the passive and electric field muscle stimulation; however, the longitudinal active properties were stronger than circumferential properties when potassium stimulation was incited.^70^ Robison et al used an inflation-extension experimental method to test the murine vagina in a biaxial configuration and overcome the limitations of other experimental methods (Figure 2).^71^ Uniaxial and planar biaxial methods require paring samples into specific shapes that do not preserve the native tissue geometry.^67,68^ Furthermore, uniaxial experiments only consider the response of the tissue in one direction and cannot fully describe the multiaxial response of tissues. The inflation-extension technique has been applied to murine arteries to describe and model the mechanical response of the tissues.^46,47,58^ Benefits of this technique include preserving native tissue geometry and interactions of the extracellular matrix, while allowing assessment of the tissue multiaxially within a physiologic range.^72-75^ Akintunde et al used the inflation-extension technique to determine and model the biomechanical properties of the nonpregnant murine vagina and to elucidate the role of elastic fibers in the mechanical response of the tissue.^50^ Mathematical parameters indicated an increase in stiffness in the high strain regime for the elastase-treated vaginal samples, suggesting that elastic fiber-collagen-fiber interactions play an important role in vaginal mechanical function.^50^ Adapting the method described by Moalli et al to test the entire vagina and supportive tissue complex in a rat model,^69^ Alperin et al investigated the impact of LOXL1 deficiency on the vagina and pelvic support tissues in the mouse.^55^ Overall, LOXL1 deficiency and subsequent disruption of elastic fiber homeostasis were correlated with a decrease in biomechanical properties of the pelvic support structures in the mouse, linking elastopathy with POP.^55^

Many experimental methods and models of mechanical properties do not account for the residual stress and strain within organs. Residual stress in soft tissue is an important biomechanical feature that has been hypothesized to contribute to the mechanical homeostasis of physiologic function.^52^ Residual stress describes the stress existing within intact tissue without external forces acting on the tissue,^52^ whereas residual strain is the deformation induced by the release of the residual stress as a result of disruption of the tissue wall.^46,52,76^ Considering female reproductive organs and estrous cycle, Capone et al quantified the differences in residual strain throughout the murine reproductive tract.^76^ The vagina, in comparison to the cervix and uterus, had the largest surface area and greatest amount of collagen. Thus, residual strain was greater in the vagina vs the cervix and uterus, suggesting a gradient of mechanical properties through the reproductive tract.^76^

Using the method developed by Moalli et al, Lowder et al investigated the dynamic biomechanical properties of the vagina and pelvic support structures during pregnancy and the postpartum period to better characterize the mechanical alterations in rat tissues.^69,77^ Tissue from pregnant rats was less stiff and exhibited increased maximum elongation compared with tissue from nonpregnant rats; however, load at failure was significantly decreased in the tissue of the gravid population. After vaginal delivery, biomechanical properties were restored in the pelvic support structures within 4 weeks postpartum.^77^ In a follow-up study using traditional uniaxial methods, Feola et al described the impact of pregnancy and vaginal delivery on the passive and active properties of the rat vagina.^78^ Vaginal distensibility increased over the time course of pregnancy as a result of the interplay of the changing passive and active properties.^78^ Intrapartum, the passive component (the extracellular matrix) decreased in tensile strength and increased in strain, with both aspects recovering in the postpartum period.^78^ Smooth muscle contractility decreased significantly over the time course of pregnancy, acting to increase distensibility, but contractility reemerged during late pregnancy.^78^ Feola et al continued to use a uniaxial tensile method to investigate the effect of parity on the vaginal mechanics in a rhesus macaque study, demonstrating a decrease in mechanical properties and a significant change of collagen fiber orientation for parous animals.^79^ Knight et al investigated the impact of parity in the ewe model and compared the results with 2 common animal models: the rhesus macaque and the rodent.^80^ The vaginal wall was stiffer and stronger in the nulliparous vs parous ewe and rhesus macaque models; however, vaginal properties did not differ with parity in the rodent model, a finding that may be attributed to a lack of maternal birth injury occurrences in the rodents.^80^ In a 2009 review, Abramowitch et al discussed the advantages and disadvantages of different animal models and biomechanical methods for investigating prolapse and concluded that further investigation is needed to determine the ideal animal model for prolapse research.^81^

While the vagina contributes to the support of the pelvic organs,^48^ and mechanical testing of the biomechanical properties of the vagina has shown that vaginal tissue is viscoelastic and anisotropic,^64,70,71^ experiments performed by Moalli et al concluded that the vagina may not be the primary source of failure in prolapse, as other pelvic support structures were implicated in these experiments*.*^69^ Furthermore, prolapsed vaginal tissue is stiffer and less elastic than nonprolapsed tissue, indicating the presence of a maladaptive remodeling process with POP.^41,65,67^ Interestingly, loss of steroidal sex hormones through oophorectomy resulted in a reduction in vaginal mechanical properties in rats compared to their nonmenopausal counterparts.^1,69^ Mechanical properties were maintained in animals treated with exogenous hormones, implicating a role for hormones in the maintenance of vaginal mechanical integrity.^1^ Additionally, in large animals that are susceptible to maternal birth injury, parity affects the mechanical properties of the vagina.^79,80^ The mechanical integrity of vaginal tissue in rhesus macaques and ewes was not recovered in the postpartum state, unlike the rat model which experienced near recovery of mechanical properties after delivery.^79,80^ A combination of factors may lead to the development of prolapse, and an improved understanding of the biomechanical properties of the vagina in both normal and altered states can enhance overall insight into the etiology of and progression to POP.

## PELVIC FLOOR LIGAMENTS

The pelvic ligaments are components of the endopelvic fascia and are crucial support structures for the pelvic organs such as the vagina, cervix, and uterus. The uterosacral ligaments and cardinal ligaments have been the focus of investigations of connective tissue failure in conjunction with prolapse.^38,39,82^ The uterosacral ligaments attach the posterior cervix and uterus to the sacrum, while the cardinal ligaments originate from the obturator fascia of the lateral pelvis and provide support to the cervix and upper vagina. The uterosacral ligaments and cardinal ligaments are composed of smooth muscle, blood vessels, nerve fibers, collagen, and elastin. Collagen and elastic fibers provide the primary structural support for these tissues.^39,83^

While the exact elastic fiber content of each ligament is unknown, premises have been established by prior studies. The uterosacral ligament is approximately 20% smooth muscle,^84^ and the deeper layers of the uterosacral ligament have more nerve innervation than the superficial layers.^85^ Comparatively, the cardinal ligament is principally composed of veins, nerves, lymphatic vessels, and loose areolar tissue.^86^ In women diagnosed with POP, both the uterosacral ligaments and cardinal ligaments have increased amounts of type III collagen.^83,84^ The role of the endopelvic fascia and pelvic ligaments in pelvic floor disorders is relatively understudied. However, research suggests the importance of these structures in maintaining the integrity of the pelvic organs and preventing prolapse.^69,81,87^

### Pelvic Floor Ligament Tissue Experimental Methods and Mechanical Properties

Uniaxial methods have been applied to investigate the mechanical properties of the pelvic ligaments.^69,88^ As mentioned previously, Moalli et al developed a method to test the entire pelvic support complex—including the vagina, pelvic floor, and pelvic ligaments—in a rat model using uniaxial tensile testing.^69^ The test imposed a constant deformation rate, in which failure of the complex was the result of paravaginal attachments rather than insult to the vaginal wall.^69^ Rivaux et al performed uniaxial experiments on uterosacral ligaments, round ligaments, and broad ligaments from female cadavers without prolapse.^88^ The goal of the study was to establish a baseline understanding of the differences in mechanical properties of 3 major ligaments in the reproductive system. The uterosacral ligament, compared to the round ligament and broad ligament, was the stiffest ligament and was hypothesized to be more critical for pelvic support.^88^

Uniaxial properties are a good initial step for understanding the basic mechanics of organs; however, biaxial testing provides more physiologic results as tissues experience multiaxial loading within the body.^67^ Thus, biaxial methods have been applied to the pelvic ligaments to better imitate the physiologic conditions through multiaxial loading. Becker and De Vita customized a protocol to investigate the biaxial stress-stretch and stress-relaxation properties of the uterosacral ligaments and cardinal ligaments in swine.^38^ They chose a swine model because of the histologic similarities between the animal and the human uterosacral and cardinal ligaments.^89^ The swine uterosacral and cardinal ligaments had an orthotropic, nonlinear response when engaged biaxially and were stiffer along the main physiologic loading direction, normal to the upper vagina or cervix. Additionally, Becker and De Vita found that the uterosacral and cardinal ligaments undergo dramatic deformations, indicating that the pelvic organs are dynamic in the pelvis.^38^ In successive research, Tan et al investigated the microstructural and biaxial creep properties of the swine uterosacral and cardinal ligaments as a complex.^39^ Using standard electron microscopy, they analyzed the extracellular matrix fiber directions. Initially, the fibers were arranged principally along the physiologic loading direction, and upon loading, straightened from a baseline crimped orientation.^39^ The arrangement of the collagen fibers along the physiologic axis could explain the increased stiffness noted in the prior study by Becker and De Vita*.*^38^ Furthermore, the creep behavior was significantly faster in the parallel direction to load but not in the perpendicular direction to load, indicating that fiber orientation and crimp play roles in the creep response. Based on these findings, future POP treatments should consider loading directions of the tissues to improve patient outcomes.^39^ In a 2016 review, Baah-Dwomoh et al compared the mechanical properties of the swine uterosacral ligament to the human uterosacral ligament and found no statistical difference in their mechanical properties.^37^ The Baah-Dwomoh et al review adds evidence that swine pelvic ligaments are an ideal model for investigating the mechanical properties of the uterosacral ligament.^37^

Because the endopelvic fascia and pelvic ligaments are key elements of pelvic organ support, failure of these structures is a potential mechanism of prolapse. Uniaxial experiments of the pelvis indicate the importance of the pelvic ligaments in determining the progression of prolapse because the pelvic ligaments and connective tissue failed before the vaginal wall.^13^ Furthermore, because the uterosacral ligament is stiffer than the cardinal and broad ligaments, the uterosacral ligament is a potentially greater contributor of support for the pelvic organs and an important clinical target.^88^ Additional research correlating the microstructure with the mechanical properties of the pelvic ligaments is needed to improve our understanding of the mechanisms of prolapse. Supporting this recommendation is the Baah-Dwomoh et al review that calls for improvement in experimental methods and in understanding of the mechanical properties of the female reproductive organs and pelvic floor.^37^

## CONCLUSION

POP is a multifactorial condition that affects a large percentage of the female population in the United States. The exact cause of prolapse remains unknown; however, researchers are working to identify and quantify the structural and functional dysfunction that may lead to the development of this condition. Understanding the structural components and biomechanical properties of tissue could improve understanding of the mechanisms behind POP. Given the limited understanding of prolapse development, more research is needed to quantify the microstructure of the pelvic organs and pelvic support structures—with and without prolapse. Additionally, identifying biomechanical properties in multiaxial configurations will improve our absolute understanding of pelvic tissue support, as well as improve our ability to establish predictive models and strive to improve clinical treatment strategies.

## Notes

This article meets the Accreditation Council for Graduate Medical Education and the American Board of Medical Specialties Maintenance of Certification competencies for Patient Care and Medical Knowledge.
